# A repeated cross‐sectional analysis of breastfeeding initiation rates in Ireland for two decades and 10 recommended priorities for improvement

**DOI:** 10.1111/mcn.13424

**Published:** 2022-09-22

**Authors:** Roy K. Philip, Aubree Worobetz, Helen Byrt, Irene Beirne, Raeghnya Zutshi, Tanya Cassidy, Colum P. Dunne

**Affiliations:** ^1^ Division of Neonatology, Department of Paediatrics University Maternity Hospital Limerick Limerick Ireland; ^2^ School of Medicine University of Limerick Limerick Ireland; ^3^ Lactation Division, Department of Nursing Midwifery University Maternity Hospital Limerick Limerick Ireland; ^4^ Neonatal Nursing, Department of Midwifery University Maternity Hospital Limerick Limerick Ireland; ^5^ Sociology and Anthropology, School of Nursing, Psychotherapy and Community Health Dublin City University Dublin Ireland; ^6^ Centre for Interventions in Infection, Inflammation and Immunity (4i) University of Limerick School of Medicine Limerick Ireland

**Keywords:** breastfeeding, health promotion, infant feeding, infant formula, lactation, policy making, population health

## Abstract

Despite a number of public health and policy‐based initiatives, Ireland's national breastfeeding rates are among the lowest globally. Regionally, the Mid‐West of Ireland has historically had low breastfeeding initiation rates, and parts of its major urban area such as Limerick City suffer the highest levels of economic deprivation in the country. In that context, this repeated cross‐sectional study analysed breastfeeding initiation trends in the Mid‐West of Ireland for two decades, from 2001 to 2020 inclusively. Statistical analysis revealed persistently low percentages of women initiating breastfeeding in the region. Time series analyses of the data demonstrated that overall breastfeeding rates are increasing, but continue to be lower than Irish national averages. From these findings and a narrative review of published research, we determined 10 plausible reasons for these consistently low breastfeeding rates. Arising from these, we propose ‘10 Priorities’ to increase the breastfeeding initiation rates in Ireland.

## Key messages

- For decades Ireland has had one of the lowest breastfeeding initiation rates globally, despite being a well‐educated European country with relatively well‐structured and supported public health policies.
- In the Mid‐West region of Ireland, overall breastfeeding rates continue to trend below national rates while concurrently housing some of the most deprived areas in the country.
- Reasons for a low breastfeeding rate encompass the social, cultural, religious, and economic contexts of Ireland.
- Improving regional and national breastfeeding rates is a complex, multi‐dimensional issue that can in part be addressed by the recommended ‘10 Priorities’ for Ireland to increase the breastfeeding rate.

## Graphical abstract

In this study, we attempted to analyse the breastfeeding initiation rates over the last 20 years for Ireland's Mid‐West region. Furthermore, we attempted to appraise the impact of deprivation in the defined city areas and, through a narrative review, proposed 10 evidence‐based themes, the ‘10 Priorities’, to improve Irish breastfeeding rates.

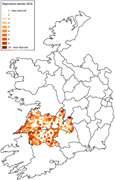

## 1 INTRODUCTION

Human milk is regarded as the ideal nutrition for newborn infants (World Health Organisation, 2003). Initiation of breastfeeding during early postpartum is pivotal for the sustenance of lactation and immune development, through the provision of dynamic and exquisite bioactive personalised nutrition (Lancet, 2016). While in some countries breastfeeding rates are over 90%, Ireland remains an outlier having one of the lowest rates in the world (Health Service Executive, 2020; World Health Organisation, 2021). A multitude of factors has been suggested to explain this societal and healthcare divergence from the anthropological norm of species‐specific feeding, arguably the most influential being the ineffectiveness of Irish public health campaigns to achieve the targets. This failure belies the ample evidence of health benefits to infant, mother, society and environment that is sufficiently convincing to most women in other countries, promoting breastfeeding as the normal nutritional choice for newborn infants. Unfortunately, in Ireland, culturally embedded practices and adherence to deep‐rooted transgenerational societal norms solidify the inertia to change and slow any beneficial impact of public health initiatives.

Despite overall reticence in Ireland, combined feeding or combination feeding (CF, breastfeeding and formula feeding [FF]) or nonexclusive breastfeeding (NEB) is an emerging trend that generally seems to impair breastfeeding initiation and sustenance. The Infant Feeding Policy for Ireland states that mothers who intend to CF should be counselled on the importance of exclusive breastfeeding (Health Service Executive, 2021). Mothers’ stated reasons for CF vary, emphasising practicalities regarding maternal sleep, inclusion of other family members and flexibility in returning to work (Hemmingway et al., 2020).

Low rates of breastfeeding could be attributed to several interconnecting factors—economic, social, cultural, religious and personal. These factors have been recognised elsewhere and frameworks exist that attempt to provide explanations, such as socioeconomic and culturally rooted models (Vanderlinden et al., 2020). Notably, despite socioeconomic factors similar to Ireland, the United Kingdom and Australia have higher breastfeeding rates, suggesting the importance of considering cultural, religious and societal norms when tailoring breastfeeding interventions (Gallegos et al., 2020).

With reference to the socioeconomic factors detailed above, Ireland's economic growth over the last three decades has not translated into a universal benefit. Areas of significant deprivation still exist and, in such areas, there is considerable potential for suboptimal levels of breastfeeding. More definitively the Trinity National Deprivation Index (TNDI) has been developed, using data collected from the Irish Census (Teljeur et al., 2019). The index is widely used in healthcare research to calculate a measure of deprivation for small geographical areas and electoral divisions in Ireland. In its most recent 2016 report, Limerick City had a few pockets of the highest rates of deprivation nationwide with over 70% of the electoral divisions in Limerick City categorised in deprived deciles. With a population of 94,192 (2016), Limerick City scored the poorest mean deprivation score nationally at 3.204 (population‐weighted). However, the TNDI denominator population for the given analysis was 51,458 with a narrow definition of Limerick city catchment resulting in an overrepresentation of inner city areas. It is worth acknowledging that areas of affluence exist along the peripheral city zones and the surrounding counties of the Mid‐West region while pockets of deprivation prevail in certain inner city electoral districts. Relevant to this study, within this same timeframe the Perinatal Statistics Report also examined Irish national levels of breastfeeding, where Limerick City was shown to have the lowest rate of breastfeeding at the time of discharge nationally (Health Research and Information Division, 2012). Perinatal statistics report of 2019 also placed the counties of Limerick and Claire among the low in the country (lowest in Donegal) for percentage distribution of exclusive breastfeeding (Healthcare Pricing Office, 2021).

In this study, we attempted to analyse the breastfeeding initiation rates over the last 20 years for Ireland's Mid‐West region with 473,269 inhabitants, or 9.94% of the Irish population (Census 2016, Central Statistics Office). Furthermore, we attempted to appraise the impact of deprivation in the defined city areas and, through a narrative review, proposed 10 evidence‐based themes, the ‘10 Priorities’, to improve Irish breastfeeding rates.

## 2 METHODS

### 2.1 Design and setting

**Figure 1:**
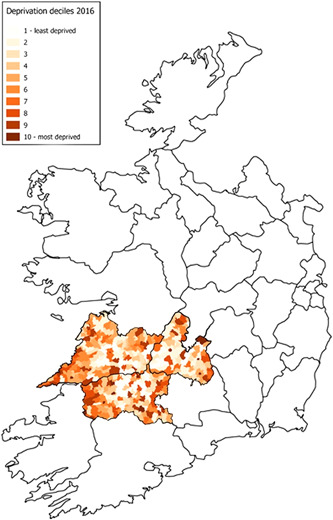
Map of national deprivation deciles for the University Maternity Hospital Limerick (UMHL) catchment area, adapted from the Trinity National Deprivation Index (Teljeur et al., 2019)

This repeated cross‐sectional observational study was performed using data collected annually over 20 years at the University Maternity Hospital Limerick (UMHL) in the Mid‐West of Ireland. UMHL is the sole provider of obstetric and neonatology services for the Mid‐West, covering one of the six designated health regions of the country. A repeated cross‐sectional design avoids overlap in each annual data set allowing valid interpretations could be made about population trends over time. The TNDI (Teljeur et al., 2019) was used to examine the deprivation index in Ireland with a focus on the catchment area for UMHL (Figure 1).

### 2.2 Population

UMHL is the only maternity hospital for a population of 473,269 from the counties of Limerick, Clare, North Tipperary and adjacent catchment areas. All infants of the region from 23 weeks of gestation are treated locally (apart from surgical or cardiac interventions). Perinatal demography and patient characteristics of the population have been published previously (Philip et al., 2018, 2020). All live births at UMHL from 1 January 2001 to 31 December 2020 were included. No mother‐infant dyads with in‐house births at UMHL were excluded and the data were not stratified based on maternal ethnicity, nationality, neighbourhood socioeconomic deprivation index, home address, socioeconomic classifications, English language proficiency, congenital anomalies of infants, multiple gestations or inconsistencies around the accuracy of gestational age estimation.

### 2.3 Procedure

Descriptive datasets were examined consisting of quantitative data collected monthly over 20 years from the labour ward register, birth notification form (BNF01), postnatal ward statistics, national perinatal epidemiology pre‐submission data, pre‐submission data to the National Baby Friendly Health Initiative (BFHI), computerised discharge compilation, neonatal unit admission register and pre‐submission data for the VON international benchmarking (Vermont Oxford Network, 2021). National data were obtained from the Economic and Social Research Institute (ESRI), Healthcare Pricing Office of Health Service Executive (HSE), Central Statistics Office (CSO) and the National Women and Infants Health Programme reports on perinatal statistics.

The main outcomes were the number of live births and the number of infants immediately commencing breastfeeding during the early postnatal period. The WHO recommends breastfeeding within the hour of birth; however, mother‐infant pairs who commenced *breastfeeding as the first feed* during any stage of postnatal stay were included as breast milk *initiation*. At discharge breastfeeding (on any day) from the hospital was included in the *discharge* data. An infant who received breast milk as the first feed and afterwards had FF during their postnatal stay was categorised CF at discharge. Similarly, if the first feed was formula and subsequently had breast milk, as per the Irish Maternity Indicator System (IMIS) the infant was still classified in receipt of CF at discharge. The date of discharge varied based on the mode of delivery (vaginal/caesarean) and the presence or absence of perinatal complications or co‐morbidities. Both at‐breast feeding and expressed breast milk (EBM) feeding were included in the overall umbrella of breastfeeding. For those infants in the neonatal unit, EBM offered via nasogastric tube was also included as breast milk intake. Additionally, data were collected on the number of infants exclusively breastfeeding (EB) and those with CF at the time of hospital discharge. Home birth‐related breastfeed and FF were not included in the analysis. Irish home birth rate is generally low. Overall planned home birth numbers in the Mid‐West region in Ireland from September 2019 to June 2020 was only 15, contributing to <0.05% of total births for the time period (Philip et al., 2020).

### 2.4 Analysis

A fully anonymized and deidentified data set fulfilling general data protection regulation (GDPR) compliance was prepared for statistical analysis (Philip, 2019) (see Supporting Information: Appendix 1). Time series analysis of the data was conducted using SPSS (v28).

### 2.5 Narrative review and search strategy

A literature search was conducted on EMBASE, PubMed, Medline, Scopus, and MedRxiv for relevant studies between 1 January 1975 to 31 December 2020. Search items in the title or abstract that included selected keywords were, ‘Ireland’, ‘breastfeeding’, ‘formula feeding’, ‘combination feeding’, ‘breastfeeding initiation’, ‘breastfeeding rate’, ‘breastfeeding + embarrassment’, ‘barrier’, ‘cultural attitudes’, ‘educational influence’, ‘antenatal education’, ‘immigration’, ‘family dynamics’, ‘vicarious experience’, ‘in‐hospital formula exposure’ and ‘advertisements’. Search was limited to peer‐reviewed English language publications of review articles, observational studies, retrospective reviews, randomised trials, systematic reviews and meta‐analysis. Opinion pieces, conference abstracts, commentaries and case reports were not included. Paired investigator agreement was required for final inclusion.

## 3 RESULTS

Between January 2001 and December 2020 there were 93,148 live births at UMHL with a mean annual inpatient birth rate of 4655 per year. The lowest was 4042 live births in 2001, to the highest of 5443 in 2008.

**Figure 2:**
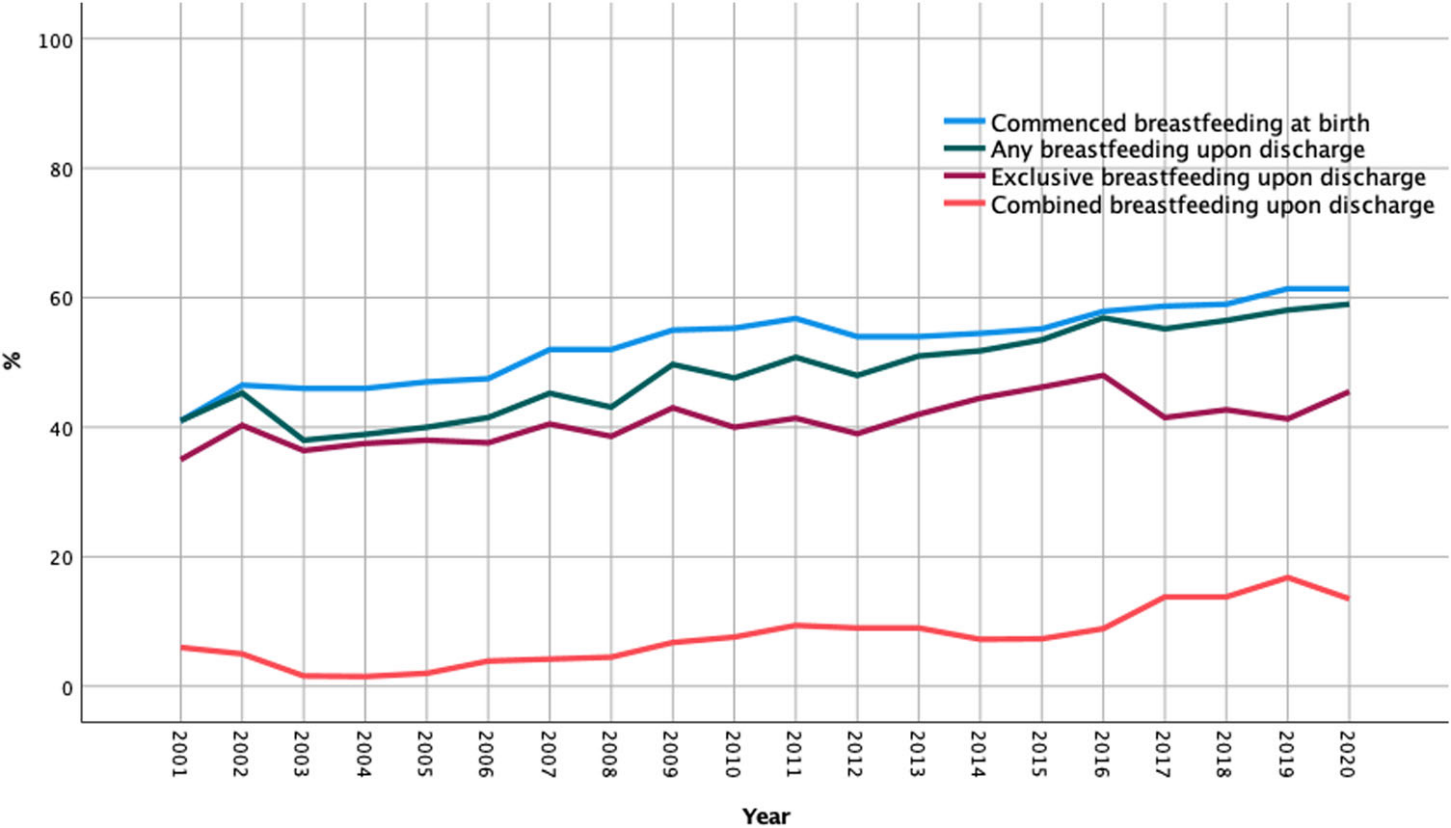
Breastfeeding rate at birth and upon discharge, University Maternity Hospital Limerick (UMHL), Mid‐West of Ireland

Overall, the number of live births that initiated breastfeeding increased from 41% in 2001 to 61.4% in 2020. Additionally, both EB and CF upon discharge from the hospital increased from 35% to 45.5% and from 6% to 13.5%, respectively. However, the percentage of infants who had any breastfeeding at discharge (either exclusive or combined) was consistently lower than the percentage that had commenced breastfeeding immediately or soon after birth, indicating discontinuation of breastfeeding during the in‐patient period (Figure 2).

**Figure 3:**
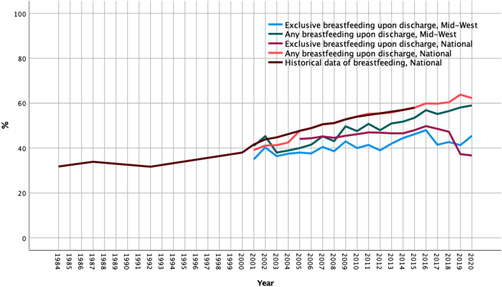
Exclusive and any breastfeeding upon discharge, Mid‐West and Nationally in Ireland, with overlying historical data (Lubold, 2019)

Irish national breastfeeding initiation rates from multiple published sources were pooled to generate data from 1984 to 2017, with certain data gaps (Baby Friendly Health Initiative in Ireland, 2017; Health Research and Information Division, 2012; Lubold, 2019; National Committee on Breastfeeding, 2005; World Health Organisation, 2021) (Figure 3). When compared to this data, *initiation rates* as well as *any breastfeeding rates* upon discharge from hospitals in the Mid‐West were lower than the national averages (Figure 3). Of note, in 2019 the national rate of EB on discharge decreased dramatically from 47.3% to 37.3% and remained low in 2020 at 36.7%. Simultaneously, the national rate of breastfeeding upon discharge increased in 2019 from 60.4% to 63.8%. However, in credit to the Mid‐West, it started with a low base of only 40% in 2005 for the region and the rate of improvement could be regarded as better than nationally achieved (starting base of 47.7% in 2005).

An abundance of research exists that examines the potential causes and reasoning behind Ireland's recurrently low breastfeeding rates. Our narrative review yielded 176 results and with duplicate exclusion and streamlining of outcome measures to the defined themes, 48 studies were deemed eligible for inclusion after a paired investigator consensus (Table 1). Potential reasons observed were summarised into five major themes and 10 subthemes: cultural issues, breastfeeding exposure, cow's milk‐based FF, advertising and prenatal education. Based on the evidence‐based themes and subthemes, ‘10 Priorities’ are proposed to improve the Irish breastfeeding rate (Table 2).

**Table 1:** Themes and subthemes relating to a low breastfeeding rate in Ireland

Theme	Subthemes	Key Point	References
Cultural issues	Cultural attitude	Negative social perception and acceptance within Irish culture is a major barrier for breastfeeding mothers.	Desmond and Meaney (2016); Komninou et al. (2017); McGorrian et al. (2010); Shortt et al. (2013); Tarrant et al. (2010)
	Perceived embarrassment	Women are not initiating breastfeeding for fear of personal embarrassment if they were to breastfeed in a public place.	Shortt et al. (2013); Tarrant et al. (2010); Connolly et al. (1998); Desmond and Meaney (2016); Scott (2011)
	Family dynamics	Reduction of exclusive breastfeeding in the twentieth century has compounding, intergenerational effects of increased formula feeding.	Rempel and Rempel (2011); Shortt et al. (2013); Wagner et al. (2006); Schafer et al. (2015); Di Manno et al. (2015); Vaz et al. (2021); Kumar et al. (2006); Pollock et al. (2002)
	Influence of immigration	The impact of a greater likelihood of breastfeeding in immigrant populations in Ireland is somewhat shadowed by the nation's cultural attitude, which has been shown to negatively impact breastfeeding behaviour in immigrant women.	Nolan and Layte (2014); H. Chen et al. (2021); Ladewig et al. (2014); Dennis et al. (2019); McGorrian et al. (2010); Castro et al. (2014); O'Sullivan et al. (2020); Brick and Nolan (2014)
Breastfeeding exposure	Child and adolescent education	Children and young adults represent an impressionable population that needs to be considered in the context of breastfeeding introduction.	McGorrian et al. (2010); Rollins et al. (2016); Shortt et al. (2013); Tarrant et al. (2010); Fewtrell et al. (2020); Connolly et al. (1998); Grant (2016); Glaser et al. (2015)
	Vicarious experience	The vicarious experience is complex with different emotions evoked and has been shown to be an important influencer for breastfeeding initiation.	Dennis (1999); Bartle and Harvey (2017); Bandura (1977); McGorrian et al. (2010); (Hoddinott et al. (2010); Hoddinott (2010)
Cows’ milk‐based formula use	In‐hospital formula exposure	Formula feeding is available immediately after birth.	Becker (2016); Chantry et al. (2014); McCoy and Heggie (2020); Brown et al. (2014)
Advertising	Lack of breastfeeding advertisements	A paucity of breastfeeding advertisement exists in comparison to formula advertisement.	Hastings et al. (2020); Godlee et al. (2019); Wolf (2007); Sobel et al. (2011)
Prenatal education	Antenatal education promotion of breastfeeding	Lack of consistency of training and knowledge.	Barnes et al. (2021); Leahy‐Warren et al. (2017); Ward et al. (2004); Finneran and Murphy (2016); Mulcahy et al. (2012); Hemmingway et al. (2020)
	Inconsistent advice	Women are receiving conflicting advice on the use and impact of formula feeding.	Kaplan and Graff (2008); Murphy et al. (2021); Desmond and Meaney (2016); O'Sullivan et al. (2020); Whelan and Kearney (2015); Basch et al. (2013)

**Table 2:** ‘Ten Priorities’ to increase the breastfeeding rate in Ireland

	Mandate	Related theme
1	Employ WHO data reporting of Irish national breastfeeding rates	Cultural issues
2	Extend entitlement to breastfeeding breaks for all breastfeeding mothers returning to work to meet the WHO standard of 2 years of age	
3	Advocate for all advantages of breastfeeding	
4	Establish cross‐cultural peer support groups	
5	Implement breastfeeding awareness and observation into primary and secondary education curriculums	Breastfeeding exposure
6	Regularise prescribing of formula milk in‐hospital	Cow's milk‐based formula use
7	Establishment of a donor human milk bank in the Republic of Ireland	
8	Institute a state‐funded advertising campaign for breastfeeding	Advertising
9	Incorporate promotion on social media in breastfeeding action plans	
10	Better staffing levels in maternity hospitals and neonatal units to support breastfeeding and every mother discharged from the hospital to be given a contact number for breastfeeding support	Prenatal education

## 4 DISCUSSION

Despite the prevailing rate of deprivation in certain areas of Limerick City since 2006, the rates of breastfeeding in the wider Mid‐West region have improved from 2001 to 2020. Although any improvement in breastfeeding is encouraging, the Mid‐West region continues to have persistently low breastfeeding initiation, mirroring the national Irish trend. During the study period, a local population shift (due to outward relocation) was observed in certain inner city ‘regeneration areas’ and this might have influenced some of the observations. The increased rate of CF observed from 6% to 13.5% has two potential causes: breastfeeding women are incorporating formula into their feeding routine or FF women are attempting to breastfeed as well. Ideally the latter would be the case; however, research has shown women who CF are more likely to cease breastfeeding over those who EB (Hemmingway et al., 2020; Holmes et al., 2011).

In general, Ireland is a well‐educated European country with well‐structured and supported public health policies aimed to enhance the overall population health. Smoking prevalence fell from 41% in 1995 to 13% in 2015 when tobacco control policies were nationally implemented (Li et al., 2018). In 2017–2018, increased awareness of the benefits of vaccination against pertussis and influenza during pregnancy was associated with increased maternal vaccine uptake in Ireland as well (Quattrocchi et al., 2019).

Irish policy and awareness specific to breastfeeding, in particular, do exist, and the lack of public health information on breastfeeding and its benefits does not seem to be an issue in Ireland (Quinn et al., 2019; Shortt et al., 2013). The HSE has implemented policies on infant feeding codes on the marketing of breastmilk substitutes (BMS) as well as a national Breastfeeding Action Plan for 2016–2021. However, it has fallen short of its goal of a 2% annual increase in breastfeeding rates (Health Service Executive, 2021).

So, the question remains—why does a population that understands the benefits of breastfeeding, and has policies devoted to its promotion, have recurrently low breastfeeding rates with such a resistance to change despite the overwhelming evidence that breastfeeding is the optimal and normal nutritional choice for the newborn infant?

### 4.1 Cultural issues

#### 4.1.1 Cultural attitude

Up until the mid‐twentieth century breastfeeding was perceived as the norm in Ireland (McGinley, 2020). During this time wet nursing was common among both upper‐ and lower‐class families, with many newborns struggling to thrive if not in the care of a wet nurse (Madden, 2021). This cultural norm shifted towards FF from the mid‐twentieth century (Cassidy, 2015; McGinley, 2020). Wet nursing and fosterage were widely practiced in Ireland for centuries and created long‐lasting ties of emotional and economic connections of ‘fictive kinship’ (Tait, 2020). Since breastfeeding suppresses ovulation, when no other contraceptive measures were used, intervals between births as noted in Parish baptism registers signalled those who ‘employed’ wet nurses and those who did not (Tait, 2020). In cultures where wet nursing was the accepted practice, the transition to FF would be different than if maternal breastfeeding was the norm. Perceived embarrassment also could differ while transitioning from maternal breastfeeding or wet nursing to FF. In 2013, a qualitative study on Irish women identified that there were perceived ‘accepted norms’ of infant feeding in their own social context that influenced their feeding choice (Shortt et al., 2013). In 2016, the major theme of a qualitative study examining barriers for working and breastfeeding mothers was the negative social perception and acceptance within Irish culture (Desmond & Meaney, 2016). Even with the use of CF, this social stigma does not seem to disappear (Komninou et al., 2017). This cultural attitude is engrained in the Irish population and is an overarching and important consideration in the reasoning behind low breastfeeding rates in Ireland.

The Traveller community (Irish Travellers) is a minority ethnic group in Ireland, with breastfeeding rates lower than the national average; concurrently, the Mid‐West of Ireland houses one of the highest numbers of traveller settlements in the country (Beirne et al., 2020). While representing 0.7% of the general population, the proportion of <14 years of age among them in 2016 was 39.7%, against the national average of 21.4% (Central Statistics Office, 2016). A multitude of factors has been postulated as the reasons for the low breastfeeding rate among this population who would benefit from culturally acceptable and targeted interventions (Beirne et al., 2020).

#### 4.1.2 Perceived embarrassment

Many Irish women highlight feelings of personal embarrassment if they were to breastfeed in a public place and quote these feelings as a principal reason for not initiating breastfeeding (Shortt et al., 2013; Tarrant et al., 2010). This feeling of embarrassment seems to be embedded at a young age, with a study of 17‐year‐old males and females also mentioning embarrassment as a perceived barrier to initiating breastfeeding (Connolly et al., 1998). It extends into the working atmosphere as well, where lack of facilities contributes to the thought that breastfeeding is not a viable option if a woman wishes to work (Desmond & Meaney, 2016). The feeling of embarrassment stems from how women perceive others will react to them when they breastfeed (Scott, 2011). While breastfeeding in public is gaining more acceptance, though still not a common sight in Ireland, milk expression—defined as removing milk from the breast manually or using a breast pump—continues to be seen globally as a distasteful bodily function to be conducted in privacy (Cohen, 2021).

#### 4.1.3 Family dynamics

Ethnography of breastfeeding narrates in relation to a reduction in wet nursing in the twentieth century, which could have contributed to the concurrent increasing rate in FF (Cassidy, 2015; Madden, 2021). Irish studies have shown a tendency of women to follow the norm that they encounter in their family surroundings (Shortt et al., 2013). This intergeneration effect has been shown to impact rates of EB, where women who were breastfed themselves tend to EB their child for longer (Di Manno et al., 2015; Vaz et al., 2021). Those who are breastfed themselves are also more likely to advise others to breastfeed—however the same is true for FF (Schafer et al., 2015). Such factors aggregating over many generations could lead to an exponential growth of intergenerational FF. As well, the negative influence of older generations on advising against breastfeeding seems to be cross‐cultural (Kumar et al., 2006).

#### 4.1.4 Influence of immigration

A systematic review and meta‐analysis demonstrated that immigrant women are more likely to breastfeed versus nonimmigrant women (Dennis et al., 2019). This is true for Ireland as well, where breastfeeding initiation rates in non‐Irish women have consistently trended higher than those of Irish‐born women (Ladewig et al., 2014; McGorrian et al., 2010). This influx of women into the country who are more likely to breastfeed is known as the ‘healthy immigrant effect’ (Chen et al., 2013; Nolan & Layte, 2014). However, the impact of a greater likelihood of breastfeeding in immigrant populations in Ireland is somewhat shadowed by the nation's cultural attitude, which has been shown to negatively impact breastfeeding behaviour in immigrant women (Nolan & Layte, 2014). The more time immigrant women spend living in Ireland, the less likely women are to maintain a high rate of breastfeeding (Castro et al., 2014; Ladewig et al., 2014). This negative effect of acculturation on breastfeeding is not unique to Ireland and is indeed seen in other countries, such as the United States of America and the United Kingdom (Bigman et al., 2018; Choudhry & Wallace, 2012). A qualitative study on Polish mothers, in particular, described Ireland's FF culture as a distinct barrier to breastfeeding (O'Sullivan et al., 2020).

A study from 2004 to 2010 examined the increase in breastfeeding rates in Ireland and also attributed it in part to an increase of non‐Irish‐born mothers (Brick & Nolan, 2014). From 2010 to 2020 there was net immigration into Ireland; in 2020, Irish nationals accounted for 28,900 (33.8%) of the 85,400 immigrants to Ireland, with the majority returning from the United Kingdom and countries within the European Union, such as Austria, Belgium, Denmark, Finland and France (Central Statistics Office, 2020). While this was the highest return of Irish since 2007, the majority of the net immigration over the study period was of non‐Irish descent. Immigration from Poland, Lithuania, Romania and Latvia to Ireland was observed in significant numbers following the expansion of the European Union on 1st May 2004. This brings to light an interesting question: what would Ireland's breastfeeding initiation rates be if not for immigration into the country? At 41.8%, births to mothers from Ireland recorded the lowest breastfeeding, while EU 15 (excluding Ireland and the United Kingdom) reported the highest proportion of breastfeeding at 72.2% (Brick & Nolan, 2014; Healthcare Pricing Office, 2019).

### 4.2 Breastfeeding exposure

#### 4.2.1 Child and adolescent education

Education on the benefits of breastfeeding is prevalent in Ireland (McGorrian et al., 2010; Rollins et al., 2016; Shortt et al., 2013). However, it is important to consider the level at which this education is offered. From an anthropological perspective, the importance of learned behaviour and cultural norms begins at a very young age, even for breastfeeding (Fewtrell et al., 2020). As a result, children and young adults represent an impressionable population in the context of a paradigm shift towards breastfeeding. Apparently innocent accessories, such as a small plastic bottle for milk, offered with a baby doll purchased for a child who then ‘practices bottle feeding’ could perhaps contribute to a perceived norm of artificial feeding in young minds, especially if they see it as the prevalent practice in family and community.

As early as 1998 health promotion strategies identified a need to educate younger men and women about breastfeeding (Connolly et al., 1998). A number of Irish breastfeeding women would like to see breastfeeding education in school, giving children and adolescents the opportunity to see it as normal behaviour, thus tying into the vicarious experience of breastfeeding. (Shortt et al., 2013). As previously mentioned, younger males and females tend to associate breastfeeding with embarrassment, with confusion between both the feeding and sexual role of the female breast (Connolly et al., 1998; Grant, 2016). Just under one‐fifth (19.2%) of infants born to mothers <20 years were exclusively breastfed (Healthcare Pricing Office, 2019). Earlier educational intervention on breastfeeding complements the view that many women who breastfeed have a positive intention to do so pre‐ or antenatally (Tarrant et al., 2010). Breastfeeding rates in Ireland also have a tendency to increase with maternal age (Healthcare Pricing Office & Health Service Executive, 2019). Although many factors influence this tendency, it highlights an important subgroup of younger individuals who may benefit from education at an earlier age, thus initiating breastfeeding earlier.

#### 4.2.2 Vicarious experience

Breastfeeding self‐efficacy is a social cognitive theory that examines how a mother perceives her own ability to breastfeed (Dennis, 1999). The idea of physically seeing breastfeeding, deemed the vicarious experience of breastfeeding, lies within this theory and has important psychological implications for women with a positive impact on their self‐efficacy to breastfeed (Bandura, 1977). The vicarious experience is complex with different emotions evoked and has been shown to be an important influencer for breastfeeding initiation (Bartle & Harvey, 2017; Hoddinott et al., 2010). The persistent culture of FF across Ireland for many decades has resulted in a loss of this vicarious experience as well as the techniques of breastfeeding, compounding the issue of nationally low breastfeeding rates (McGorrian et al., 2010).

### 4.3 Cows’ milk‐based formula use

#### 4.3.1 In‐hospital formula exposure

In the first half of the twentieth‐century Irish breastfeeding rates were high. The introduction of FF in the late 1960s correlated to a drastic drop in these rates, to as low as 10% EB in some hospitals (Becker, 2016). In Ireland, the Infant Feeding Policy outlines that breastfed newborns should not be given any other food or fluids other than breastmilk unless medically indicated (Health Service Executive, 2021). When supplemented with formula in hospitals, women are at a very high risk of stopping breastfeeding, alongside a risk of interference with maternal milk production and suckling behaviours (Brown, 2015; Chantry et al., 2014).

Very few Irish hospital policies on breastfeeding in maternity units explicitly state principles in keeping with the aim of EB. A study of all 15 policies in Ireland revealed only one policy stating that no free infant formula would be provided to mothers on discharge from the hospital, and only three policies stated that the sale of formula was prohibited on the hospital premises (McGorrian et al., 2010). Additionally, the formula continues to be free in the hospital, while a breastfeeding mother often hires pumping equipment at her own expense (McGorrian et al., 2010).

### 4.4 Advertising

#### 4.4.1 Lack of breastfeeding advertisements

The unfortunate reality is that promotion and advertisement have turned formula milk and BMS, what should be specialised foods, into normal and accepted foods for any infant. Media plays a strong role in the choice of FF, with the BMS industry dominated by a small number of powerful corporations (Hastings et al., 2020).

Recent reports from the WHO and United Nations highlight that infant formula makers are still breaking the rules through ‘pervasive, aggressive and misleading’ marketing campaigns targeting women and health workers in many countries (United Nations, 2022; World Health Organisation, 2022). Ireland is not immune to this global marketing issue, especially when the country is a main global exporter of cow's milk‐based infant formula (O'Byrne, 2021). There are restrictions imposed by organisations (such as the Food Safety Authority of Ireland and the Baby Feeding Law Group) that attempt to regulate the advertising of BMS in Ireland, often citing the WHO's International Code of Marketing of BMS as the foundation of these regulations (Baby Feeding Law Group Ireland & IBFAN Ireland, 2015; Food Safety Authority of Ireland, 2020). In March 2019, the British Medical Journal (BMJ) made the decision to stop carrying advertisements for any BMS in their journals, citing the evidence of the global harm to health caused by aggressive promotion of BMS products (Godlee et al., 2019).

In Ireland, the first television advertisement for the promotion of breastfeeding was not aired until 2000 and was funded by the La Leche League of Ireland—not by the State. Over the last two decades attempts to advertise breastfeeding in Ireland are negligible and have been overshadowed by the mass media of BMS advertisement and policy. Despite decades of low breastfeeding rates, regulating BMS advertisements in Ireland continues to be at the forefront of marketing policy, while employing marketing interventions and investing in the promotion of breastfeeding continues to take a backseat. As part of national campaigns on breastfeeding promotion Ireland requires greater implementation of the WHO code of advertising and perhaps a ban on all advertising of BMS.

### 4.5 Prenatal education

#### 4.5.1 Antenatal education promotion of breastfeeding

It is important to highlight impactful moments in the timeline of breastfeeding education and awareness where breastfeeding information and support are crucial. Antenatal appointments are important for both screening and assessing the needs of women and their decision regarding infant feeding (Barnes et al., 2021). The decision to breastfeed is often made early in pregnancy (Leahy‐Warren et al., 2017; Ward et al., 2004) and effective breastfeeding support throughout all stages of pregnancy is essential to counter the negative cultural views in Ireland (Leahy‐Warren et al., 2017). There is an opportunity to ensure that antenatal appointments are standardised to provide adequate breastfeeding support (Mulcahy et al., 2012). Research has identified fathers as an important source of support in the decision to breastfeed and its implementation as well (Bar‐Yam & Darby, 1997). One Irish study highlighted that just over half of the fathers attended at least one antenatal class and only one‐third felt the value of shared decision‐making with their partner regarding breastfeeding (Kenosi et al., 2011).

A study in 2016 examined the attitudes of general practitioners (GPs) in the Mid‐West towards breastfeeding. Although almost 100% of GPs acknowledged the benefit of breastfeeding only 80% promoted breastfeeding within their practice, citing time and perceived cultural negativity as barriers (Finneran & Murphy, 2016). The same study also identified that only 10% of the GPs had received formal training in breastfeeding issues.

#### 4.5.2 Inconsistent advice

Information that women receive during their prenatal care plays an influential role in the decision to breastfeed (Kaplan & Graff, 2008). Although evidence for the benefits of breastfeeding is overwhelming there are still inconsistencies in messaging about infant feeding. A qualitative study of over 3000 Irish women in 2021 who indicated a barrier to breastfeeding was receiving conflicting advice from healthcare professionals regarding FF (Murphy et al., 2021). This barrier has been seen in other Irish qualitative studies as well (Desmond & Meaney, 2016; O'Sullivan et al., 2020; Whelan & Kearney, 2015). This conflicting advice is compounded by the effects of BMS marketing, mentioned above (Basch et al., 2013).

In general, improving regional and national breastfeeding rates is a complex, multi‐dimensional issue. Based on our study results, known deprivation rates in Ireland, cultural trends and a narrative review of literature, we have outlined ‘10 Priorities’ for Ireland to increase the breastfeeding rates. Each priority has been associated with an identified theme, as summarised in Table 2, and detailed in Supporting Information: Appendix 2.

Hospital policies on breastfeeding exist for good reason and need to be enforced. However, these policies are not positioned to be ‘anti‐formula feeding’, and neither should the regularisation of prescribing cows’ milk‐based formula milk. It is important to recognise the choice that exists for the mother to decide on EB, FF or CF for her newborn infant. Some women and men will inevitably choose to FF; it is the role of healthcare services and professionals to ensure this choice is not made as a result of sheer convenience, unregulated access to cows’ milk‐based formula, or lack of hospital policy adherence. On the other hand, if women do not have a clinical indication for use of cows’ milk‐based formula, free in‐hospital supply of the same by the State also merits a review.

An entire focus on the promotion of breastfeeding is missing, and in the context of a world concerned with climate change and its effects, is a subject that needs greater consideration in the rhetoric surrounding breastfeeding. Wherever an FF advertisement exists, a breastfeeding advertisement—covering the far‐reaching benefits—should not be far behind it.

### 4.6 Limitations

The authors acknowledge the following limitations of this study: (1) The TNDI has not been stratified for different socioeconomic statuses; (2) Catchment area of UMHL contains a mixed profile of deprivation indexes; (3) Breastfeeding data are generalised and not stratified for confounding factors such as maternal illness or maternal characteristics or social class; (4) Home birth‐related breastfeeding and FF were not included in the analysis, as the Irish home birth rate is <1% (Central Statistics Office, 2020), and data on breastfeeding initiation/exclusive or combination were not available for the region for two decades; (5) cost–benefit evaluation was not discussed in the ‘10 Priorities’; (6) Some of the historical data defines ‘breastfeeding rate’, ‘breastfeeding initiation rate’ and ‘exclusive breastfeeding rate’ with a non‐rigorous definition, thus posing difficulties for direct comparison.

## 5 CONCLUSION

The time has come to break through the cultural barrier to breastfeeding that exists in Ireland. As an educated, high‐income European country there are no excuses for a breastfeeding rate that, for decades, has been among the lowest in the world. As laid out in the ‘10 Priorities’, this paradigm shift should begin with the younger generations and persist among policies, organisational endeavours and community collaboration. These priorities identified could change the conversation about breastfeeding and propel a culture that engages in and encourages breastfeeding, empowering mothers, families and healthcare providers to ensure that all babies in Ireland are given the option to feed in the most natural, beneficial, economical and environmentally sustainable way available.

## AUTHOR CONTRIBUTIONS

Roy K. Philip conceptualised and designed the study, extracted the data spanning two decades, structured the scientific content, reviewed and revised the manuscript. Aubree Worobetz prepared and revised the manuscript, developed the tables and figures and incorporated references, edits and formatting. Helen Byrt contributed to the data collection and expert knowledge. Irene Beirne and Tanya Cassidy contributed expert knowledge of the research topic. Raeghnya Zutshi contributed to the initial draught development. Colum P. Dunne advised on methodology and manuscript structure and critically reviewed the manuscript for important intellectual content. All authors read and approved the final manuscript as submitted and agree to be accountable for the work. The corresponding author attests that all listed authors meet the authorship criteria.

## CONFLICT OF INTEREST

The authors declare no conflict of interest.

## ETHICS STATEMENT

University Hospital Limerick Research Ethics Committee approval was granted for the study.

## Supporting information

Supporting information.

Supporting information.

## Data Availability

All relevant data are included in the manuscript. Fully anonymized and deidentified data set fulfilling general data protection regulation (GDPR) compliance is provided as a supplementary file (Supporting Information: Appendix 1).
