# Supplementary material for: A repeated cross‐sectional analysis of breastfeeding initiation rates in Ireland for two decades and 10 recommended priorities for improvement
Source: Matern Child Nutr. 2022 Sep 22;19(1):e13424. doi: 10.1111/mcn.13424 (PMC9749595; doi:10.1111/mcn.13424)
Supplement: Supplementary file 1 — Supporting information. [file MCN-19-e13424-s002.docx]

**Supplementary Appendix 1: Datasets**

Table 1 Initial data, collected, 2001-2020

| Year | Live births | Commenced breastfeeding | | Exclusive breastfeeding upon discharge | | Combined feeding upon discharge | | Any breastfeeding upon discharge | |
| --- | --- | --- | --- | --- | --- | --- | --- | --- | --- |
|  |  | Newborns | % | Newborns | % | Newborns | % | Newborns | % |
| 2001 | 4042 | 1657 | 41 | 1415 | 35 | 243 | 6 | 1658 | 41 |
| 2002 | 4371 | 2033 | 46.5 | 1762 | 40.3 | 219 | 5 | 1981 | 45.3 |
| 2003 | 4514 | 2076 | 46 | 1643 | 36.4 | 72 | 1.6 | 1715 | 38 |
| 2004 | 4418 | 2032 | 46 | 1657 | 37.5 | 66 | 1.5 | 1723 | 38.9 |
| 2005 | 4411 | 2073 | 47 | 1676 | 38 | 88 | 2 | 1764 | 40 |
| 2006 | 4692 | 2229 | 47.5 | 1764 | 37.6 | 183 | 3.9 | 1947 | 41.5 |
| 2007 | 5153 | 2680 | 52 | 2087 | 40.5 | 216 | 4.2 | 2303 | 45.25 |
| 2008 | 5443 | 2830 | 52 | 2101 | 38.6 | 245 | 4.5 | 2346 | 43.1 |
| 2009 | 5432 | 2988 | 55 | 2336 | 43 | 368 | 6.76 | 2708 | 49.7 |
| 2010 | 5233 | 3003 | 55.3 | 2173 | 40 | 413 | 7.6 | 2586 | 47.6 |
| 2011 | 5137 | 2918 | 56.8 | 2127 | 41.4 | 483 | 9.4 | 2610 | 50.8 |
| 2012 | 4905 | 2649 | 54 | 1913 | 39 | 441 | 9 | 2354 | 48 |
| 2013 | 4594 | 2481 | 54 | 1929 | 42 | 413 | 9 | 2342 | 51 |
| 2014 | 4522 | 2464 | 54.5 | 2012 | 44.5 | 328 | 7.25 | 2340 | 51.8 |
| 2015 | 4690 | 2588 | 55.2 | 2167 | 46.2 | 344 | 7.33 | 2511 | 53.5 |
| 2016 | 4474 | 2590 | 57.9 | 2148 | 48 | 398 | 8.9 | 2546 | 56.9 |
| 2017 | 4416 | 2592 | 58.7 | 1833 | 41.5 | 609 | 13.8 | 2442 | 55.2 |
| 2018 | 4439 | 2619 | 59 | 1895 | 42.7 | 613 | 13.8 | 2508 | 56.5 |
| 2019 | 4144 | 2544 | 61.4 | 1711 | 41.3 | 696 | 16.8 | 2407 | 58.1 |
| 2020 | 4118 | 2528 | 61.4 | 1874 | 45.5 | 556 | 13.5 | 2430 | 59 |

Table 2 National data, collated, 1984-2020

| Year | Live births | Exclusive breastfeeding upon discharge [%] | Any breastfeeding upon discharge [%] |
| --- | --- | --- | --- |
| 1984^†^ |  | -- | 31.8 |
| 1985 |  | -- | -- |
| 1986 |  | -- | -- |
| 1987^†^ |  | -- | 33.9 |
| 1988 |  | -- | -- |
| 1989 |  | -- | -- |
| 1990 |  | -- | -- |
| 1991 |  | -- | -- |
| 1992^†^ |  | -- | 31.7 |
| 1993 |  | -- | -- |
| 1994 |  | -- | -- |
| 1995 |  | -- | -- |
| 1996 |  | -- | -- |
| 1997 |  | -- | -- |
| 1998 |  | -- | -- |
| 1999 |  | -- | -- |
| 2000 |  | -- | -- |
| 2001^‡^ | 57922 | -- | 39.1 |
| 2002^‡^ | 60522 | -- | 41.1 |
| 2003^‡^ | 61632 | -- | 41.3 |
| 2004^‡^ | 62067 | -- | 42.5 |
| 2005^§^ | 61786 | 44.1 | 47.7 |
| 2006^§^ | 65810 | 44.4 | 48.9 |
| 2007^§^ | 71963 | 45.2 | 50.6 |
| 2008^§^ | 75587 | 44.6 | 51.1 |
| 2009^§^ | 76023 | 45.5 | 52.8 |
| 2010^§^ | 75600 | 46.2 | 54.1 |
| 2011^§^ | 74377 | 47 | 55.3 |
| 2012^§^ | 71986 | 46.9 | 55.4 |
| 2013^§^ | 69267 | 46.6 | 55.9 |
| 2014^§^ | 67610 | 46.6 | 57 |
| 2015^§^ | 65869 | 48 | 58 |
| 2016^3§^ | 64097 | 49.8 | 59.9 |
| 2017^§^ | 62070 | 48.6 | 59.8 |
| 2018^§^ | 61084 | 47.3 | 60.4 |
| 2019^¶^ | 59123 | 37.3 | 63.8 |
| 2020^¶^ | 56607 | 36.7 | 62.3 |

^†^Data extracted from (Lubold, 2019)

^‡^Data extracted from ESRI Report on Perinatal Statistics

^§^Data extracted from Perinatal Statistics Report through Healthcare Pricing Office

^¶^Data extracted from Irish Maternity Indicator System National Report
