# Supplementary material for: A repeated cross‐sectional analysis of breastfeeding initiation rates in Ireland for two decades and 10 recommended priorities for improvement
Source: Matern Child Nutr. 2022 Sep 22;19(1):e13424. doi: 10.1111/mcn.13424 (PMC9749595; doi:10.1111/mcn.13424)
Supplement: Supplementary file 2 — Supporting information. [file MCN-19-e13424-s001.docx]

**Supplementary Appendix 2: ‘Ten Priorities’ for improving breastfeeding rate in Ireland.**

## **Employ WHO data reporting of Irish national breastfeeding rates.**

The WHO is not only a major policy influencer on breastfeeding and related initiatives, but a facilitator for data collection on breastfeeding globally. Unfortunately, continuous national data for Ireland in this powerful database is lacking, with only two years of data (2009, 2010) for infants breastfed at three months of age. Bolstering the collection of national data would help understand the gravity of the breastfeeding situation in Ireland, monitor and understand breastfeeding trends, and create a central data source for national statistics. This will in-turn support investigation and research on the trends compared to other European health statistics.

## **Extend entitlement to breastfeeding breaks for all breastfeeding mothers returning to work to meet the WHO standard of two years of age.**

Although Ireland’s paid maternity leave is comparable to other European countries, new mothers attempting to EB still struggle to breastfeed upon returning to work, often taking holiday or unpaid leave in order to meet the WHO guidelines (Desmond & Meaney, 2016). The Maternity Protection Act (Amended) 2004 allows breastfeeding breaks for up to 26 weeks after birth, which although aligns with the WHO recommendations of EB until six months of age, falls short of supporting mothers to continue breastfeeding up to two years of age. Encouraging and facilitating women to continue to breastfeed after they return to work will help to normalise breastfeeding within Irish culture and promote continued breastfeeding as a viable option for working mothers (Desmond & Meaney, 2016). The HSE’s implementation of breastfeeding breaks at work for up to one hour per normal working day, in addition to normal rest breaks, until the child’s second birthday is a step in the right direction.

## **Advocate for all advantages of breastfeeding.**

Further to the health effects, the economic and environmental advantages of breastfeeding over FF are indisputable and of growing concern globally (Rollins et al., 2016; Zadkovic, Lombardo, & Cole, 2020). Breastfeeding uses fewer resources and produces minimal to no waste, in contrast to FF which exacerbates environmental damage (Joffe, Webster, & Shenker, 2019). In 2021 an Irish collaborative research demonstrated that the emissions saved from achieving an exclusive breastfeeding rate of 50% for six months would be greater if renewable energy (decarbonising) was opted instead of fossil fuel in the production of breastmilk substitutes (Long et al., 2021). Additionally, La Leche League of Ireland, alongside government initiatives such as Healthy Ireland, have acknowledged these environmental issues are of growing concern in Ireland. Previous research in Ireland and abroad has demonstrated that women from the lower socioeconomic status are less likely to breastfeed (Ladewig, Hayes, Browne, Layte, & Reulbach, 2014; Pontin, Emmett, Steer, & Emond, 2007). This seems counterintuitive to the vast economical and financial benefits of breastfeeding, and represents an undervalued avenue in the promotion of breastfeeding. Financial incentives to breastfeed are also a current trend in research but have significant socio-economic, ethical, and moral implications (Hoskins & Schmidt, 2021; Relton et al., 2018).

## **Establish cross cultural peer support groups.**

The WHO recommends peer support interventions for initiating and continuing breastfeeding. Additionally, peer support interventions have been found to support minority and hard to reach groups in healthcare (Fisher et al., 2015). Notably, in the United Kingdom (UK), there are organizations commissioned to deliver breastfeeding peer support groups specifically to areas of socio-economic deprivation (Hunt, Thomson, Whittaker, & Dykes, 2021). Importantly, interest in breastfeeding support intervention is high in Ireland (Alberdi et al., 2018). Peer support groups have proved effective in increasing breastfeeding rates and are a prime opportunity for women to increase their confidence in breastfeeding and gain a psycho-social support network (Ingram, 2013; Kaunonen, Hannula, & Tarkka, 2012). In the context of breastfeeding there has been limited improvement in addressing the cultural differences encountered by immigrant mothers in Ireland (Chen et al., 2021). The national Breastfeeding Action Plan in Ireland 2016 to 2021 pays very little attention to the potential influence of non-Irish born mothers. The specific inclusion of minority and immigrant groups in peer support groups would not only bridge the cultural gap, but also support the social exposure of Irish-born women – who historically favor FF – with immigrant women who conversely tend to breastfeed.

## **Implement breastfeeding awareness and observation into primary and secondary education curriculums.**

The idea of incorporating promotion of breastfeeding into the Irish education curriculum is not new – in fact junior cycle students in Irish secondary schools receive a breastfeeding information pack, produced by the Department of Health. However, providing written information on breastfeeding and its benefits to young adults is not sufficient enough to instill a wave of change onto future Irish generations. The normalization of breastfeeding must begin at young age, from picture books with breastfeeding mothers, to the vicarious experience of secondary school students who have the opportunity to see, firsthand, how breastfeeding looks in reality. The constraints to this priority are of course the social implications. However, this perceived constraint highlights why curriculums need to enforce visual and real representation of breastfeeding.

## **Regularize prescribing of formula milk in-hospital.**

In 2016 the Health Research Board published a report indicating avoiding supplementary infant feeding is important to improve breastfeeding rates in Ireland (Sutton, O'Donoghue, Keane, Farragher, & Long, 2016). In-hospital cows’ milk-based formula use continues to be a major barrier to breastfeeding in Ireland, despite the Infant Feeding Policy stating that breastfed newborns should not be given any other food or fluids other than breastmilk unless medically indicated (Health Service Executive, 2021). The provision of free of charge cows’ milk-based formula in the medical environment has ethical implications that warrant a revision at the national level.

The use of in-hospital cows’ milk-based formula requires stricter regulation. Therefore, if there is a neonatal or maternal contraindication for breastfeeding (such as maternal illness, maternal medications of concern, structural breast abnormalities, any structural or functional facial-maxillary concern in the infant, suspected inborn error of metabolism for infant, insufficient supply despite appropriate lactation support, or mental health issues) and human donor milk is not available, the indication should be documented prior to prescribing and beginning any cows’ milk-based formula feeding.

## **Establishment of a donor human milk bank in the Republic of Ireland.**

When maternal milk is not available or insufficient, use of pasteurised donor human milk (DHM) has proved invaluable and is encouraged by the European Society for Paediatric Gastroenterology, Hepatology and Nutrition, as well as by the WHO (World Health Organization, 2011). The use of DHM is especially important in the context of preterm infants and normalising human milk feeding (Sánchez Luna, Martin, & Gómez-de-Orgaz, 2021). Currently a single human milk bank located in Northern Ireland supplies DHM to the Island of Ireland and in total received 1229.7 litres of DHM from 266 donors and supplied the 21 units in the Republic of Ireland (ROI) in 2019 (19 maternity units and two paediatric intensive care units catering for surgical neonates) with support of the voluntary charitable assistance from Blood Banks Ireland for the timely transport (Power, O’Dea, & O’Grady, 2019).

Establishing a milk bank in the ROI requires capital investment and an in-depth analysis of the multistep process associated with donating, storing, pasteurising and supplying DHM (Biasini et al., 2013). The UK Association of Milk Banking (UKAMB) is a national organization dedicated to the support and promote milk banking (UK Association of Milk Banking, 2021), however no equivalent organization currently exists in Ireland. As well, the European Milk Bank Association is a non-profit organization that promotes milk banking throughout Europe, and acknowledges lack of a milk bank in the Republic of Ireland (compared to thirteen milk banks in England alone) (European Milk Bank Association, 2021). Although detailed European-wide guidelines on DHM banks are lacking (Weaver et al., 2019), Ireland has the benefit of an existing DHM bank in Northern Ireland with an established process and foundation to learn from. We must be proactive in this matter and address the ‘true banking crisis in Ireland’, to support the increasing number of Irish mothers and neonates who rely on DHM for their health and wellbeing. While working arrangements are currently in place for DHM to reach neonatal units in the ROI even after the decision of the UK to leave the European Union, a change in European or UK law could considerably strain the access of neonatal units to DHM. While Brexit, the formal departure of United Kingdom from the European Union (EU), was formally declared on 31^st^ January 2021, working arrangements are in place for the unhindered supply of DHM from the milk bank stationed in Northern Ireland (part of United Kingdom) to the maternity hospitals and recipients in the Republic of Ireland, south of the boarder (part of European Union). However, it leaves one to speculation as to how the differences between EU and UK laws in relation human milk or cells and tissues might influence the free movement of DHM across the border between UK and EU in the future. Self reliance through the development of a human milk bank in the Republic of Ireland would offer mitigation against the uncertainties to a great extent.

## **Institute a State-funded advertising campaign for breastfeeding.**

The most recent national Breastfeeding Action Plan 2016 to 2021 has made no mention of State-funded advertising for breastfeeding. Time and resources are continually funnelled into restricting and regulating BMS marketing, yet the promotion of breastfeeding itself on the same public channels is non-existent. After decades of the BMS market dominating the media it is time to recognize the importance of formulating an opposition, and leveling the advertising field with breastfeeding advertisements that are reinforced by the State.

## **Incorporate promotion on social media in breastfeeding action plans.**

An important challenge that Ireland faces is the need for a cultural shift in favor of breastfeeding. The influence of social media on breastfeeding has been an under-utilized resource in Ireland, and in fact has considerable merit in the context of health communication campaigns (Snyder, 2007). Instagram, among other social media platforms, offers an opportunity to help normalize breastfeeding with its diverse, global community (Marcon, Bieber, & Azad, 2019). Studies have previously identified that breastfeeding mothers use social media for education, advice and social support (Tomfohrde & Reinke, 2016), and campaigns for breastfeeding in other countries have shown success (Sriraman & Kellams, 2016). As the HSE Action Plan for Breastfeeding 2016 to 2021 comes to an end there is an opportunity to incorporate social media initiatives and campaigns into future action plans, and take advantage of these influential, global platforms.

## **Better staffing levels in maternity hospitals and neonatal units to support breastfeeding and every mother discharged from hospital to be given a contact number for breastfeeding support.**

Lactation consultants work within both the public and private setting within healthcare in Ireland and are invaluable beyond their interactions with breastfeeding mothers; lactation consultants are perceived to be a central point of contact for health professionals as well as for training, problem case management, and referrals (Dunne & Fallon, 2020; McGorrian, Shortt, Doyle, Kilroe, & Kelliher, 2010). In Ireland, one-on-one consultations have been viewed as one of the most helpful interventions postnatally to successfully engage in breastfeeding (Alberdi et al., 2018). In general, lactation consultants are consistently found to be important influencers in the breastfeeding process (Brown, Dodds, Legge, Bryanton, & Semenic, 2014; Whelan & Kearney, 2015).

UMHL is one of the few maternity units in the country that provide seven-day specialized breastfeeding support – ideally this would be offered in all maternity units in Ireland. In 2021 the Irish government announced newly allocated funding for 24 additional lactation consultant positions across Ireland as part of the national Breastfeeding Action Plan 2016 to 2021 helping to address a shortage of lactation consultants in the country (Department of Health, 2021; Murphy, Rohde, Foley, O'Carroll, & Flynn, 2021). It is worth acknowledging initiatives such as the live web chat via [www.mychild.ie](http://www.mychild.ie) . This online support is operational five days a week and offers lactation consultant support for breastfeeding after hospital discharge. In addition to information provided by the HSE on [www.hse.ie](http://www.hse.ie), other resource locations for breastfeeding support in the community include, La Leche League of Ireland via [www.lalecheleagueireland.com](http://www.lalecheleagueireland.com), the Association of Lactation Consultants in Ireland via www.alcireland.ie, Irish Childbirth Trust (Cuidiu) via [www.cuidiu.ie](http://www.cuidiu.ie) and, Safe Food and Healthy Ireland via [www.makeastart.ie](http://www.makeastart.ie)

Alberdi, G., O'Sullivan, E. J., Scully, H., Kelly, N., Kincaid, R., Murtagh, R., . . . McAuliffe, F. M. (2018). A feasibility study of a multidimensional breastfeeding-support intervention in Ireland. *Midwifery, 58*, 86-92. doi:<https://doi.org/10.1016/j.midw.2017.12.018>

Biasini, A., Stella, M., Malaigia, L., China, M., Azzalli, M., Laguardia, M. C., & Rizzo, V. (2013). Establishment, operation and development of a donor human milk bank. *Early Human Development, 89*, S7-S9. doi:<https://doi.org/10.1016/j.earlhumdev.2013.07.005>

Brown, C. R. L., Dodds, L., Legge, A., Bryanton, J., & Semenic, S. (2014). Factors influencing the reasons why mothers stop breastfeeding. *Canadian Journal of Public Health, 105*(3), e179-e185. doi:10.17269/cjph.105.4244

Chen, H., Li, C., Zhou, Q., Cassidy, T. M., Younger, K. M., Shen, S., & Kearney, J. M. (2021). How to promote exclusive breastfeeding in Ireland: a qualitative study on views of Chinese immigrant mothers. *International Breastfeeding Journal, 16*(1), 10. doi:10.1186/s13006-021-00358-4

Department of Health. (2021). Budget 2022 delivering additional €31 million for women's health [Press release]. Retrieved from <https://www.gov.ie/en/press-release/309be-budget-2022-delivering-additional-31-million-for-womens-health/>)

Desmond, D., & Meaney, S. (2016). A qualitative study investigating the barriers to returning to work for breastfeeding mothers in Ireland. *International Breastfeeding Journal, 11*(1), 16. doi:10.1186/s13006-016-0075-8

Dunne, S., & Fallon, A. (2020). Public health nurses' experiences of supporting women to breastfeed in community settings in Ireland. *Journal of Health Visiting, 8*(6), 240-246. doi:10.12968/johv.2020.8.6.240

European Milk Bank Association. (2021). About EMBA - United Kingdom. Retrieved from <https://europeanmilkbanking.com/country/united-kingdom/>

Fisher, E. B., Ayala, G. X., Ibarra, L., Cherrington, A. L., Elder, J. P., Tang, T. S., . . . Simmons, D. (2015). Contributions of peer support to health, health care, and prevention: papers from peers for progress. *The Annals of Family Medicine, 13*(Suppl 1), S2. doi:10.1370/afm.1852

Health Service Executive. (2021). Policies and Guidelines - Breastfeeding. Retrieved from <https://www.hse.ie/eng/about/who/healthwellbeing/our-priority-programmes/child-health-and-wellbeing/breastfeeding-healthy-childhood-programme/policies-and-guidelines-breastfeeding/>

Hoskins, K., & Schmidt, H. (2021). Breastfeeding, personal responsibility and financial incentives. *Public Health Ethics*. doi:10.1093/phe/phab020

Hunt, L., Thomson, G., Whittaker, K., & Dykes, F. (2021). Adapting breastfeeding support in areas of socio-economic deprivation: a case study approach. *International Journal for Equity in Health, 20*(1), 83. doi:10.1186/s12939-021-01393-7

Ingram, J. (2013). A mixed methods evaluation of peer support in Bristol, UK: mothers’, midwives’ and peer supporters’ views and the effects on breastfeeding. *BMC Pregnancy and Childbirth, 13*(1), 192. doi:10.1186/1471-2393-13-192

Joffe, N., Webster, F., & Shenker, N. (2019). Support for breastfeeding is an environmental imperative. *BMJ, 367*, l5646. doi:10.1136/bmj.l5646

Kaunonen, M., Hannula, L., & Tarkka, M.-T. (2012). A systematic review of peer support interventions for breastfeeding. *Journal of Clinical Nursing, 21*(13-14), 1943-1954. doi:<https://doi.org/10.1111/j.1365-2702.2012.04071.x>

Ladewig, E. L., Hayes, C., Browne, J., Layte, R., & Reulbach, U. (2014). The influence of ethnicity on breastfeeding rates in Ireland: a cross-sectional study. *Journal of Epidemiology and Community Health, 68*(4), 356. doi:10.1136/jech-2013-202735

Long, A., Mintz-Woo, K., Daly, H., O'Connell, M., Smyth, B., & Murphy, J. D. (2021). Infant feeding and the energy transition: a comparison between decarbonising breastmilk substitutes with renewable gas and achieving the global nutrition target for breastfeeding. *Journal of Cleaner Production, 324*, 129280. doi:<https://doi.org/10.1016/j.jclepro.2021.129280>

Marcon, A. R., Bieber, M., & Azad, M. B. (2019). Protecting, promoting, and supporting breastfeeding on Instagram. *Maternal & Child Nutrition, 15*(1), e12658. doi:<https://doi.org/10.1111/mcn.12658>

McGorrian, C., Shortt, E., Doyle, O., Kilroe, J., & Kelliher, C. (2010). *“Breastfeeding is natural, but it’s not the norm in Ireland”: an assessment of the barriers to breastfeeding and the service needs of families and communities in Ireland with low breastfeeding rates*. Retrieved from Dublin, Ireland: <https://www.hse.ie/eng/about/who/healthwellbeing/our-priority-programmes/child-health-and-wellbeing/breastfeeding-healthy-childhood-programme/research-and-reports-breastfeeding/barriers-to-breasfteeding-ucd-report-2010.pdf>

Murphy, R., Rohde, D., Foley, C., O'Carroll, T., & Flynn, R. (2021). This study aimed to explore women's experiences of infant feeding in Ireland. *European Journal of Public Health, 31*.

Pontin, D., Emmett, P., Steer, C., & Emond, A. (2007). Patterns of breastfeeding in a UK longitudinal cohort study. *Maternal & Child Nutrition, 3*(1), 2-9. doi:<https://doi.org/10.1111/j.1740-8709.2007.00062.x>

Power, B. D., O’Dea, M. I., & O’Grady, M. J. (2019). Donor human milk use in neonatal units: practice and opinions in the Republic of Ireland. *Irish Journal of Medical Science, 188*(2), 601-605. doi:10.1007/s11845-018-1873-3

Relton, C., Strong, M., Thomas, K. J., Whelan, B., Walters, S. J., Burrows, J., . . . Renfrew, M. J. (2018). Effect of financial incentives on breastfeeding: a cluster randomized clinical trial. *JAMA Pediatrics, 172*(2), e174523-e174523. doi:10.1001/jamapediatrics.2017.4523

Rollins, N. C., Bhandari, N., Hajeebhoy, N., Horton, S., Lutter, C. K., Martines, J. C., . . . Victora, C. G. (2016). Why invest, and what it will take to improve breastfeeding practices? *The Lancet, 387*(10017), 491-504. doi:<https://doi.org/10.1016/S0140-6736(15)01044-2>

Snyder, L. B. (2007). Health communication campaigns and their impact on behavior. *Journal of Nutrition Education and Behavior, 39*(2, Supplement), S32-S40. doi:<https://doi.org/10.1016/j.jneb.2006.09.004>

Sriraman, N. K., & Kellams, A. (2016). Breastfeeding: what are the barriers? Why women struggle to achieve their goals. *Journal of Women's Health, 25*(7), 714-722. doi:10.1089/jwh.2014.5059

Sutton, M., O'Donoghue, E., Keane, M., Farragher, L., & Long, J. (2016). *Interventions that promote increased breastfeeding rates and breastfeeding duration among women: an umbrella review*. Retrieved from Dublin: <https://www.hrb.ie/fileadmin/publications_files/Interventions_that_promote_increased_breastfeeding_rates_2016.pdf>

Sánchez Luna, M., Martin, S. C., & Gómez-de-Orgaz, C. S. (2021). Human milk bank and personalized nutrition in the NICU: a narrative review. *European Journal of Pediatrics, 180*(5), 1327-1333. doi:10.1007/s00431-020-03887-y

Tomfohrde, O. J., & Reinke, J. S. (2016). Breastfeeding mothers' use of technology while breastfeeding. *Computers in Human Behavior, 64*, 556-561. doi:<https://doi.org/10.1016/j.chb.2016.07.057>

UK Association of Milk Banking. (2021). About UKAMB. Retrieved from <https://ukamb.org/about/>

Weaver, G., Bertino, E., Gebauer, C., Grovslien, A., Mileusnic-Milenovic, R., Arslanoglu, S., . . . Picaud, J.-C. (2019). Recommendations for the establishment and operation of human milk banks in Europe: a consensus statement from the European Milk Bank Association (EMBA). *Frontiers in Pediatrics, 7*(53). doi:10.3389/fped.2019.00053

Whelan, B., & Kearney, J. M. (2015). Breast-feeding support in Ireland: a qualitative study of health-care professionals’ and women’s views. *Public Health Nutrition, 18*(12), 2274-2282. doi:10.1017/S1368980014002626

World Health Organization. (2011). *Guidelines on Optimal Feeding of Low Birthweight Infants in Low and Middle-income Countries*. Retrieved from Geneva:

Zadkovic, S., Lombardo, N., & Cole, D. C. (2020). Breastfeeding and climate change: overlapping vulnerabilities and integrating responses. *Journal of Human Lactation, 37*(2), 323-330. doi:10.1177/0890334420920223
